# Peptide inhibition of acute lung injury in a novel two-hit rat model

**DOI:** 10.1371/journal.pone.0259133

**Published:** 2021-10-28

**Authors:** Alana C. Sampson, Brittany P. Lassiter, Magdielis Gregory Rivera, Pamela S. Hair, Kaitlyn G. Jackson, Adrianne I. Enos, Turaj Vazifedan, Alice L. Werner, Marshall J. Glesby, Frank A. Lattanzio, Kenji M. Cunnion, Neel K. Krishna

**Affiliations:** 1 ReAlta Life Sciences, Norfolk, Virginia, United States of America; 2 Department of Microbiology, Eastern Virginia Medical School, Norfolk, Virginia, United States of America; 3 Department of Pediatrics, Eastern Virginia Medical School, Norfolk, Virginia, United States of America; 4 Children’s Hospital of The King’s Daughters, Norfolk, Virginia, United States of America; 5 Children’s Specialty Group, Norfolk, Virginia, United States of America; 6 Weill Department of Medicine, Weill Cornell Medicine, New York, New York, United States of America; 7 Department of Physiological Sciences, Eastern Virginia Medical School, Norfolk, Virginia, United States of America; University of Messina, ITALY

## Abstract

Acute lung injury (ALI) often causes severe trauma that may progress to significant morbidity and mortality. ALI results from a combination of the underlying clinical condition of the patient (e.g., inflammation) with a secondary insult such as viral pneumonia or a blood transfusion. While the secondary insult may be variable, the rapidly progressive disease process leading to pulmonary failure is typically mediated by an overwhelming innate immunological or inflammatory reaction driven by excessive complement and neutrophil-mediated inflammatory responses. We recently developed a ‘two-hit’ ALI rat model mediated by lipopolysaccharide followed by transfusion of incompatible human erythrocytes resulting in complement activation, neutrophil-mediated ALI and free DNA in the blood indicative of neutrophil extracellular trap formation. The objective of this study was to evaluate the role of peptide inhibitor of complement C1 (RLS-0071), a classical complement pathway inhibitor and neutrophil modulator in this animal model. Adolescent male Wistar rats were infused with lipopolysaccharide followed by transfusion of incompatible erythrocytes in the presence or absence of RLS-0071. Blood was collected at various time points to assess complement C5a levels, free DNA and cytokines in isolated plasma. Four hours following erythrocyte transfusion, lung tissue was recovered and assayed for ALI by histology. Compared to animals not receiving RLS-0071, lungs of animals treated with a single dose of RLS-0071 showed significant reduction in ALI as well as reduced levels of C5a, free DNA and inflammatory cytokines in the blood. These results demonstrate that RLS-0071 can modulate neutrophil-mediated ALI in this novel rat model.

## Introduction

Acute lung injury (ALI) is often a complication of severe trauma that can progress to acute respiratory distress syndrome (ARDS) resulting in significant morbidity and mortality [1]. To date, there are no pharmacological interventions to prevent ALI with current standard of care being supportive in nature. ALI may result from a combination of the underlying clinical condition of the patient (e.g., inflammation, trauma, hypotension) with a secondary insult such as a blood transfusion (transfusion-related ALI (TRALI), resuscitation, radiation) [2–4] or viral pneumonia (e.g., influenza, respiratory syncytial virus or coronavirus-related ALI) [5–7]. While the secondary insult may differ, the rapidly progressive disease process leading to pulmonary failure is typically mediated by an exaggerated and overwhelming innate immunological or inflammatory response driven by excessive complement and neutrophil-mediated inflammatory responses. In addition to ALI, dysregulated neutrophil and complement activation are key mediators of acute exacerbations in chronic lung diseases such as COPD and steroid resistant neutrophilic asthma [8, 9].

In the case of TRALI, which represents one of the leading causes of transfusion-related mortality, this disease process is complex and not fully understood, however a ‘two-hit’ model is currently believed to most accurately exemplify the clinical situation with the first hit mediated by the underlying clinical condition of the patient and the second hit triggered by a component in the transfused unit [10, 11]. Various in vitro, in vivo and ex vivo studies have implicated neutrophils as a key player in the pathogenesis of TRALI through direct activation, formation of reactive oxygen species (ROS) and neutrophil extracellular trap (NET) formation resulting in acute lung injury (ALI) [12]. Additionally, it has previously been postulated that the complement system may play a role in TRALI through C3a and C5a interaction with neutrophils resulting in neutrophil activation as well as ROS and NET formation [13], however studies showing such a direct connection have to date been lacking.

Our laboratory has recently developed a novel two-hit rat model of ALI in which Wistar rats are infused with lipopolysaccharide (LPS) to stimulate neutrophils followed 30 minutes later with a transfusion of 30% incompatible erythrocytes to activate the classical complement pathway [14]. Four hours after transfusion, animals are neutropenic as reported in TRALI patients [2] and histology of the lung tissue reveals massive neutrophil infiltration of the lung parenchyma. Additionally, a time course over the four-hour period demonstrates significant increases in complement C5a levels as well as free DNA in the blood. Free DNA is indicative of NET formation and has been demonstrated to be a biomarker for TRALI in both mouse models and in humans [15, 16].

Over the last few years, our laboratory has developed a novel family of anti-inflammatory molecules known as Peptide Inhibitors of Complement C1 (PIC1) that inhibit the classical pathway of complement inhibit neutrophil-mediated myeloperoxidase (MPO) and NET activity [17–20] and exhibit antioxidant and antimicrobial activity [21, 22]. The lead compound, RLS-0071, consists of a 15 amino acid peptide with the sequence IALILEPICCQERAA with a 24-mer monodisperse PEG moiety fused to the C terminus to improve solubility [17]. Given the ability of this peptide to modulate complement and neutrophil activity, the purpose of this study was to assess the efficacy of RLS-0071 in this novel model of complement-induced, neutrophil-mediated ALI. Our results demonstrate that RLS-0071 given as a one-time prophylactic or rescue dose regimen can mitigate neutrophil and inflammatory cytokine mediated ALI in this model.

## Materials and methods

### Ethics statement and animal welfare

Animal Research: Animal research was approved by the Eastern Virginia Medical School (EVMS) IACUC. For euthanasia of rats, animals deeply anesthetized with a cocktail of ketamine/acepromazine were subsequently subject to isoflurane inhalation followed by decapitation by guillotine. Adolescent male Wistar rats (200–250 g) were purchased from Hilltop Lab Animals (Scottdale, PA, USA) with indwelling jugular catheters. Care and handling of the animals were in accord with NIH guidelines.

Human subjects research: Human subjects research was approved by the EVMS IRB, protocol #02-06-EX 0216. Written consent was obtained. A healthy human volunteer (type AB+) donating whole blood was used as the source of purified human erythrocytes.

### Human erythrocyte purification

Human erythrocytes from an AB+ donor were acquired the day before the animal experiments and processed as described previously [23]. Briefly, 20 mL of human blood was purified on a Histopaque (Sigma-Aldrich, Saint Louis, MO, USA) gradient by centrifugation. The erythrocytes were then separated from white blood cells and platelets and resuspended in saline. Rats (200g) have a nominal circulating blood volume of 14 mL with a nominal 40% hematocrit. For transfusion, 2 mL of human erythrocytes at 80% hematocrit was administered, which results in a 30% transfusion to the rats. To exclude the possibility that human granulocytes were present in the erythrocyte preparations, the purified erythrocyte preparations were analyzed for contaminating granulocytes on a hemocytometer. Visual inspection of multiple fields of erythrocyte preparations with final cell counts of 8 x 10^10^ cells/ml did not reveal any viable contaminating human granulocytes.

### Animal experiments

**Fig 1:**
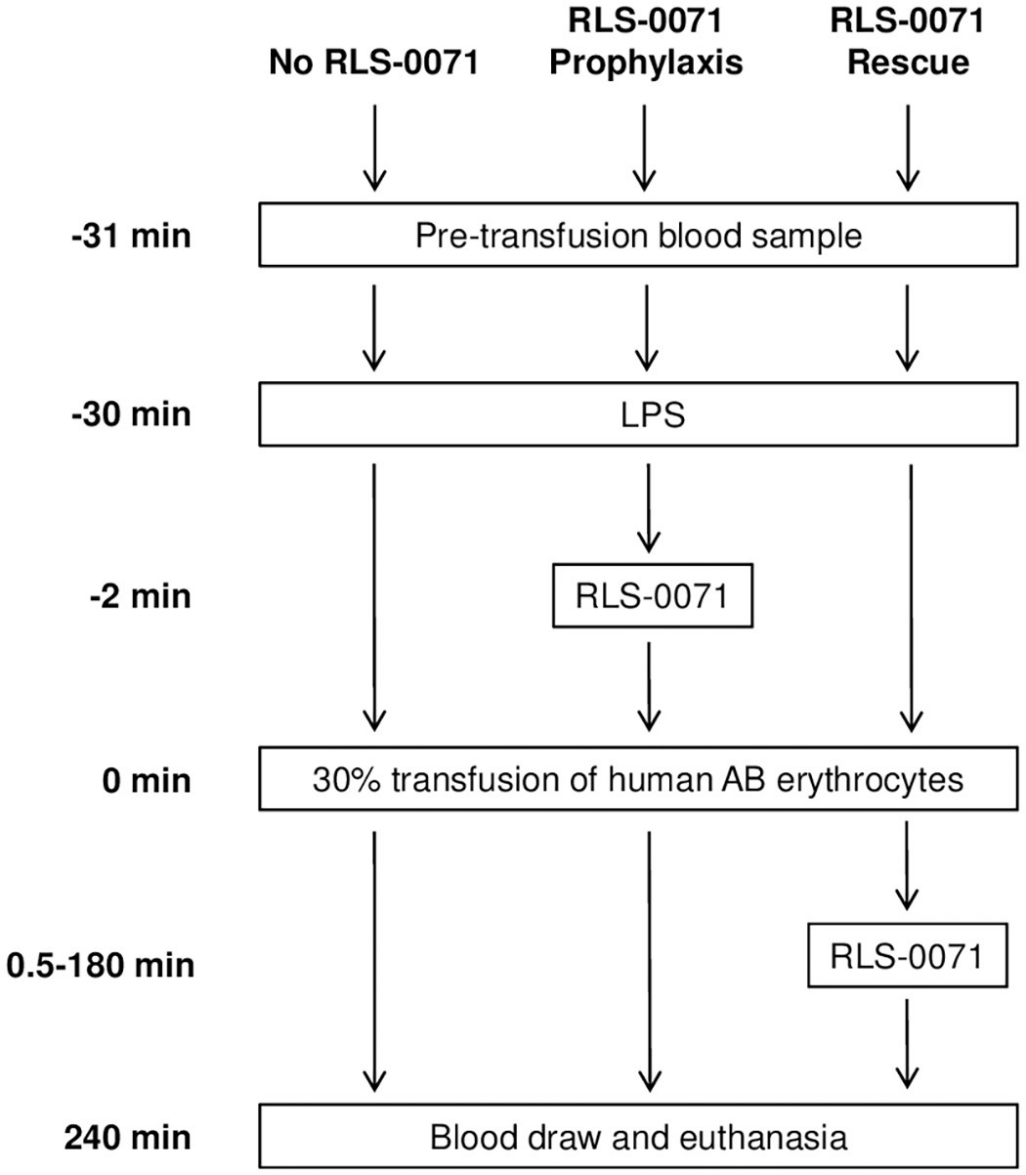
Experimental design and study arms.

Establishment of the ALI model was previously reported [21]. For all procedures, rats were sedated with ketamine (McKesson, Las Colinas, TX, USA) and acepromazine (Patterson Veterinary, Saint Paul, MN, USA) at (75/2.5 mg/kg IP) throughout the course of the experiment with monitoring of vital signs. Animals were allowed to wake up between blood draws and were re-sedated before the terminal blood draw. Lipopolysaccharide (LPS, from *Salmonella enterica* serotype enteritidis, 2 mg/kg (1 mL total volume) [MilliporeSigma, Burlington, MA, USA]) was administered intravascularly through the indwelling jugular catheter as the ‘first-hit’. This was followed 30 minutes later by 30% ABO mismatched erythrocyte transfusion as the ‘second-hit’ (Fig 1). Sham animals and animals receiving the LPS first hit only were used as controls. For RLS-0071 treated animals, the peptide consisting of the amino acid sequence IALILEPICCQERAA with a monodisperse, 24mer polyethylene glycol (PEG) tail, was manufactured by PolyPeptide Group (San Diego, CA) to ≥ 95% purity as verified by HPLC and mass spectrometry analysis. Lyophilized RLS-0071 was solubilized in 0.05 M Histidine buffer and pH adjusted to 6.5. RLS-0071 was administered as single doses of 10, 40 or 160 mg/kg to animals 28 minutes after LPS administration for prophylactic dosing and at various times after the incompatible erythrocyte transfusion as a rescue dose at 40mg/kg (Fig 1).

Blood samples were collected prior to LPS or erythrocyte administration (time 0) and at 5, 60, and 240 minutes after erythrocyte transfusion (Fig 1). Plasma was isolated from the blood and analyzed for C5a levels, free DNA concentration and cytokines levels as described below. Upon completion of the final blood draw, the sedated animals were euthanized using isoflurane (McKesson) and guillotine. A necropsy was completed to collect organs for histopathology.

### Lung injury score

Scoring of lung injury was performed by the following methodology: fixed lung tissue was stained with hematoxylin and eosin (H&E) and images were visualized with a microscope (BX50, Olympus) at a magnification of 20X at room temperature. Images of randomized microscopy fields were acquired with a digital camera (DP70, Olympus) and were converted to black and white images and the pixels were quantified by ImageJ (NIH) analysis. The ratio of black to white pixels was then determined. A minimum of 10 images were analyzed per slide for each animal.

### Plasma C5a measurements

C5a levels were measured from plasma samples by ELISA, according to the manufacturer’s instructions (LSBio, Seattle, WA) as previously described [14]. Briefly, diluted rat plasma samples were added to wells pre-coated with C5a antibodies. The plate was incubated for 90 minutes at 37°C, then any unbound components were removed by washing. One hundred μL of a biotin-conjugated C5a detection antibody (diluted 1:100) was added and incubated for 60 minutes at 37°C. Next, 100 μL of streptavidin-horseradish peroxidase (HRP) conjugate (diluted 1:100) was added and incubated for 30 minutes at 37°C. Following washing, 90 μL of 3,3’,5,5’-tetramethylbenzidine (TMB) substrate was added for 30 minutes at 37°C. The reaction was stopped with the addition of 50 μL of sulfuric acid and absorbance measured at 450 nm using a BioTek microplate reader. Sample concentrations were calculated using a C5a standard curve made with 1:2 serial dilutions.

### Plasma DNA measurements

Free DNA was measured by PicoGreen in rat plasma samples as previously described [19]. Briefly, plasma samples were diluted in 10 mM Tris-HCl, 1 mM EDTA, pH 8.0 (TE) buffer and 50uL of each sample was added to the wells along with 50uL of a 1:200 dilution of PicoGreen (Life Technologies, Carlsbad, CA, USA) and incubated at room temperature for 10 minutes, protected from light. A DNA standard curve was prepared in TE buffer. The fluorescence was then read at an excitation wavelength of 485nm and an emission wavelength of 520nm using a BioTek microplate reader. All free DNA measurements were done in triplicate.

### Cytokine analysis

Plasma samples were analyzed for cytokines and chemokines by the Cytokine Core (Indianapolis, IN, USA) using xMAP bead-based immunoassay technology specific for rat. Plasma samples were analyzed for the following experimental groups: sham, 1-hit, 2-hit, 2-hit + 10 mg/kg prophylactic dose RLS-0071, 2-hit + 160 mg/kg prophylactic dose RLS-0071, 2-hit + 40 mg/kg rescue dose RLS-0071 at 30 seconds, and 2-hit + 160 mg/kg rescue dose RLS-0071 at 30 seconds. For each cytokine reported, two replicates were run for each animal. Data are means and standard error of the means.

### Statistical analysis

Data are represented as mean and standard error of the mean. A Generalized Linear Model was used for analyzing the level of lung damage between experimental groups. The comparisons between experimental groups in C5a, DNA, and cytokines were conducted using bootstrap approach or Welch’s ANOVA. The multiple comparisons in C5a and DNA were two-sided and analyzed by using bootstrap approach and Games-Howell methods. The comparisons in cytokines variables were one-sided using 2-hit as the control group and performed using Dunnett’s test. All statistical tests were performed using R 3.5.2 and SPSS 26 (Chicago, IL). P value ≤ 0.05 was considered statistically significant.

## Results

### RLS-0071 reduces neutrophil-mediated ALI

**Fig 2:**
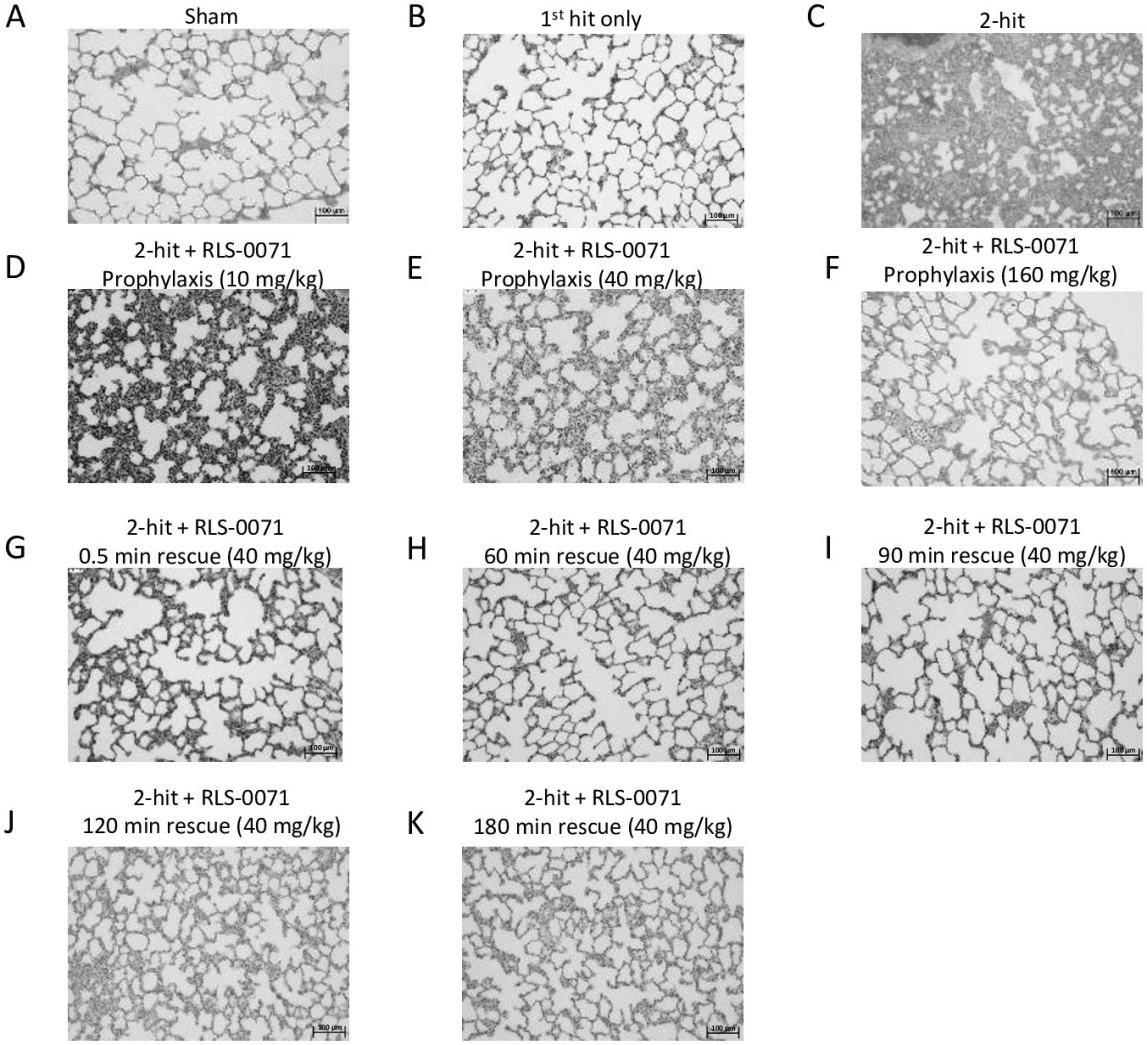
Prophylactic or rescue dosing of RLS-0071 reduces acute lung injury. Representative histology (H&E stain) of rat lungs. (A) sham control, (B) first hit only, (C) 2-hit, (D) 2-hit + 10 mg/kg prophylactic dose RLS-0071, (E) 2-hit + 40 mg/kg prophylactic dose RLS-0071, (F) 2-hit + 160 mg/kg prophylactic dose RLS-0071, (G) 2-hit + 40 mg/kg rescue dose RLS-0071 at 0.5 min, (H) 2-hit + 40 mg/kg rescue dose RLS-0071 at 60 min, (I) 2-hit + 40 mg/kg rescue dose RLS-0071 at 90 min, (J) 2-hit + 40 mg/kg rescue dose RLS-0071 at 120 min and (K) 2-hit + 40 mg/kg rescue dose RLS-0071 at 180 min. Bar represents 100 μm. Tissues were observed with a microscope (BX50, Olympus) at a magnification of 20X at room temperature. Images were acquired with a digital camera (DP70, Olympus).

Our previously developed two-hit ALI model is initiated by infusion of LPS (first hit) into Wistar rats followed 30 minutes later with transfusion of 30% incompatible erythrocytes (second hit) and sacrifice of the animals 4 hours later [14]. Lungs of the animals show dramatic neutrophil-mediated ALI as well as robust complement activation and NETosis as measured by C5a levels and free DNA in the bloodstream, respectively. RLS-0071 is a dual targeting anti-inflammatory molecule that inhibits both classical complement pathway activation and NETosis [17–20]. To evaluate the ability of RLS-0071 to mitigate lung damage in this model, animals were treated with a single prophylactic dose of RLS-0071 administered 2 minutes prior to the second hit or as a rescue dose at various times after the second hit (Fig 1). Lungs were isolated from animals four hours after the second hit and tissues evaluated by H&E staining. As previously described [21], sham animals (Fig 2A) or animals receiving the first hit of LPS alone (Fig 2B) displayed normal lung tissue architecture whereas animals that received the 2-hit insult showed striking lung damage mediated by substantial neutrophil infiltration into the alveolar walls (Fig 2C). In contrast, animals receiving prophylactic doses of RLS-0071 at 10, 40 or 160 mg/kg 2 minutes before incompatible erythrocyte transfusion showed a marked reduction in lung damage with the lung tissue showing lung morphology similar to that of sham animals (Fig 2D–2F). Animals receiving rescue dosing of 40 mg/kg RLS-0071 at 0.5, 60, 90, 120 and 180 after administration of the second hit also displayed lung tissue architecture resembling that of sham animals (Fig 2G–2K).

**Fig 3:**
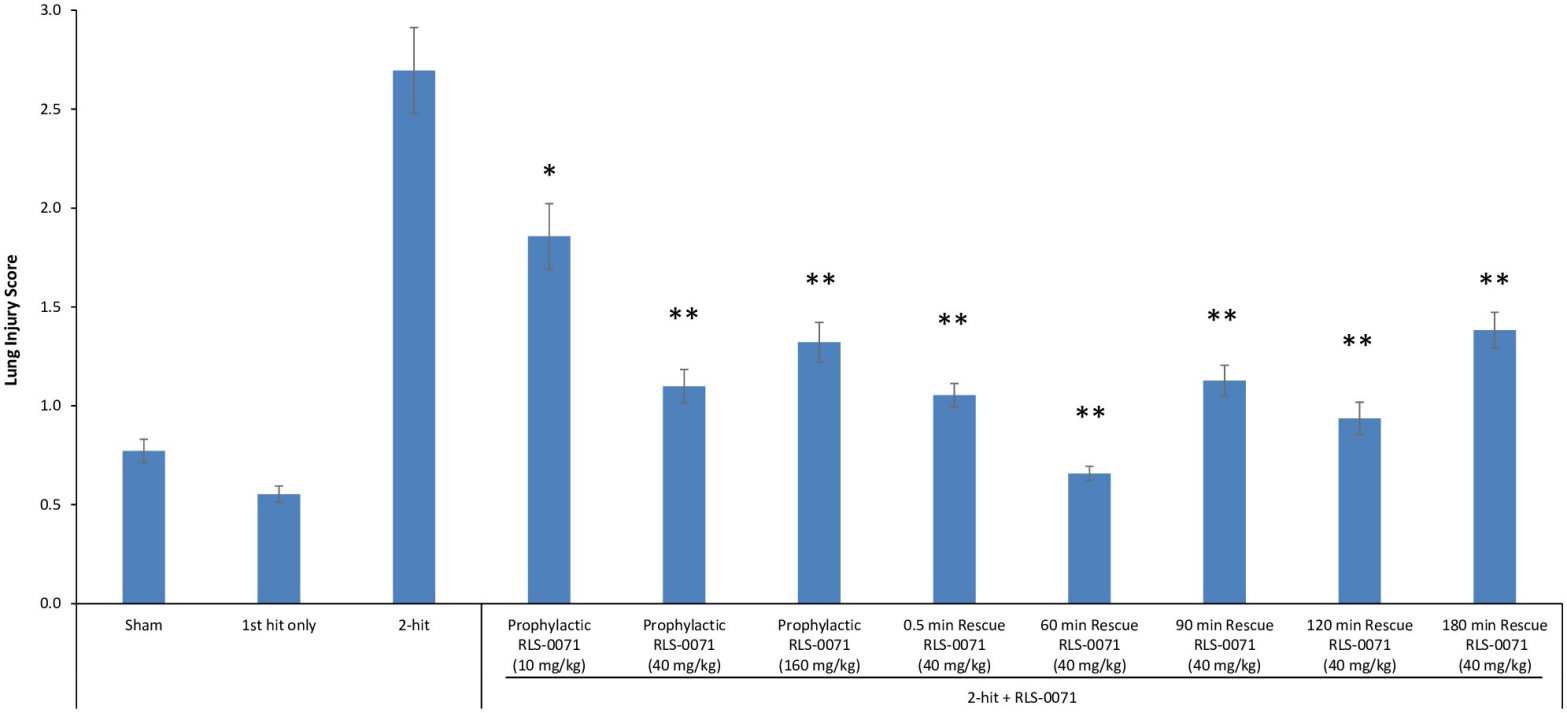
Prophylactic or rescue dosing of RLS-0071 reduces neutrophil-mediated lung injury. H&E stained lung tissue images were converted to black and white and quantified by ImageJ analysis. The ratio of black to white pixels was calculated and used as a measure of lung injury (Y axis). Sham control animals (n = 3), first hit only (n = 2), 2-hit (n = 3), 2-hit + 10 mg/kg prophylactic dose RLS-0071 (n = 4), 2-hit + 40 mg/kg prophylactic dose RLS-0071 (n = 6), 2-hit + 160 mg/kg prophylactic dose RLS-0071 (n = 9), 2-hit + 40 mg/kg rescue dose RLS-0071 at 0.5 min (n = 4), 2-hit + 40 mg/kg rescue dose RLS-0071 at 60 min (n = 3), 2-hit + 40 mg/kg rescue dose RLS-0071 at 90 min (n = 5), 2-hit + 40 mg/kg rescue dose RLS-0071 at 120 min (n = 3) and 2-hit + 40 mg/kg rescue dose RLS-0071 at 180 min (n = 3). Ten images or more were quantified per slide for each animal. Data are means and standard error of the means. Statistical analysis was performed using a Generalized Linear Model. * denotes p = 0.002 and ** denotes p<0.001 compared to 2-hit animals.

To determine the level of lung tissue protection by RLS-0071 in this model, grading of H&E sections for cell wall thickening for the different treatment groups was performed. Images of randomized microscopy fields were converted to black and white and quantified by ImageJ (NIH) analysis. The ratio of black to white pixels was then determined as a measure of lung damage: as lung damage increases, the alveolar walls thicken, shrinking the alveolar space (white space), resulting in a decrease in white pixels and increase in black pixels. Consistent with the lack of tissue damage directly visualized by microscopic observation of the H&E sections, sham animals and animals receiving the LPS first-hit only had low lung injury scores whereas animals receiving the 2-hit insult demonstrated a much higher injury score as previously demonstrated (Fig 3A) [21]. Animals receiving a 10mg/kg prophylactic dose of RLS-0071 showed significant reduction in lung damage (p = 0.002) and this effect was enhanced in animals receiving a 40mg/kg prophylactic dose (p<0.001) compared to untreated 2-hit animals. Rats prophylactically dosed at 160 mg/kg of RLS-0071 had a similar lung score as the 40mg/kg dose indicating that dosing beyond 40 mg/kg did not offer any additional protection to the lung tissue (p = 0.33 comparing the 40mg/kg and 160mg/kg doses) (Fig 3A). To evaluate if dosing animals at various times after the second hit could mitigate lung damage, animals subject to the two-hit insult were treated with 40 mg/kg RLS-0071 at 0.5, 60, 90, 120 and 180 minutes after the erythrocyte transfusion. Treatment with RLS-0071 at all time points after the second hit demonstrated significant reduction in lung damage (all p<0.001) (Fig 3B). These results suggest that a single dose of RLS-0071 can significantly attenuate acute lung injury in this experimental model up to 3 hours after the 2-hit insult.

### RLS-0071 reduces C5a production in the blood

**Fig 4:**
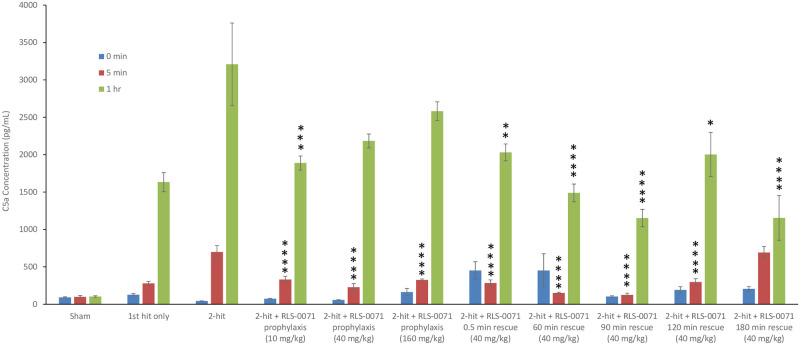
RLS-0071 inhibits complement activation. Plasma was isolated from sham animals (n = 3) and the following groups prior to first-hit (0 minutes) and at 5 minutes and 1 hour: first hit only (n = 3), 2-hit (n = 3), 2-hit + 10 mg/kg prophylactic dose RLS-0071 (n = 8), 2-hit + 40 mg/kg prophylactic dose RLS-0071 (n = 4), 2-hit + 160 mg/kg prophylactic dose RLS-0071 (n = 5), 2-hit + 40 mg/kg rescue dose RLS-0071 at 0.5 min (n = 5), 2-hit + 40 mg/kg rescue dose RLS-0071 at 60 min (n = 3), 2-hit + 40 mg/kg rescue dose RLS-0071 at 90 min (n = 5), 2-hit + 40 mg/kg rescue dose RLS-0071 at 120 min (n = 3) and 2-hit + 40 mg/kg rescue dose RLS-0071 at 180 min (n = 3). C5a was then measured in each sample by ELISA and absorbance was read at 450 nm. Two replicates for each animal were measured for every time point. Data are means and standard error of the mean. Statistical analysis was performed using were conducted using bootstrap approach or Welch’s ANOVA. * denotes p = 0.010, ** denotes p = 0.004, *** denotes p = 0.002, and **** denotes p≤0.001 compared to 2-hit animals.

We had previously demonstrated that the complement system is activated in this rat model as measured by blood levels of C5a taken at various time points after the second hit [21]. Animals receiving the first-hit of LPS only display increased levels of C5a which is attributed to LPS-mediated alternative pathway activation whereas animals receiving the 2-hit insult demonstrate much higher levels of C5a due to the combination of alternative pathway activation via LPS and classical pathway mediated activation via the incompatible erythrocyte transfusion [21, 23, 24]. To evaluate the effect of RLS-0071 on C5a production in this model, rats subject to the 2-hit insult were treated with prophylactic or rescue doses of RLS-0071 and C5a levels measured from blood samples taken at 0, 5 minutes and 1 hour after the second-hit insult. As previously demonstrated [21], sham animals had baseline levels of C5a production whereas animals receiving the LPS first-hit showed increasing levels at 5 minutes and 1 hour (Fig 4). Animals receiving the 2-hit insult had substantially more C5a production at the 1-hour time point as expected (Fig 4). Animals receiving RLS-0071 as prophylactic doses of 10, 40 and 160 mg/kg showed significant reduction of C5a at the 5 minute time point for each dose group (p<0.001) and with the exception of the 180 minute rescue dose, reduction of C5a at the 1 hour time points with the 10mg/kg dose reaching significance (p = 0.002) (Fig 4). As observed with prophylactic dosing, at the 5 minute time point, the 40 mg/kg rescue doses of RLS-0071 administered at 0.5 (p = 0.001), 60 (p<0.001), 90 (p<0.001) and 120 (p = 0.001) minutes after the 2-hit insult also demonstrated significantly decreased levels of C5a with the exception of animals receiving the rescue dose at 180 minutes (Fig 4). At the 1 hour time point, all rescue doses had significantly reduced levels of C5a compared to the 2-hit only animals (0.5 (p = 0.004), 60 (p<0.001), 90 (p<0.001), 120 (p = 0.010) and 180 (p<0.001) minutes. These findings demonstrate that RLS-0071 can significantly inhibit complement activation in this model.

### RLS-0071 inhibits free DNA accumulation in the blood

**Fig 5:**
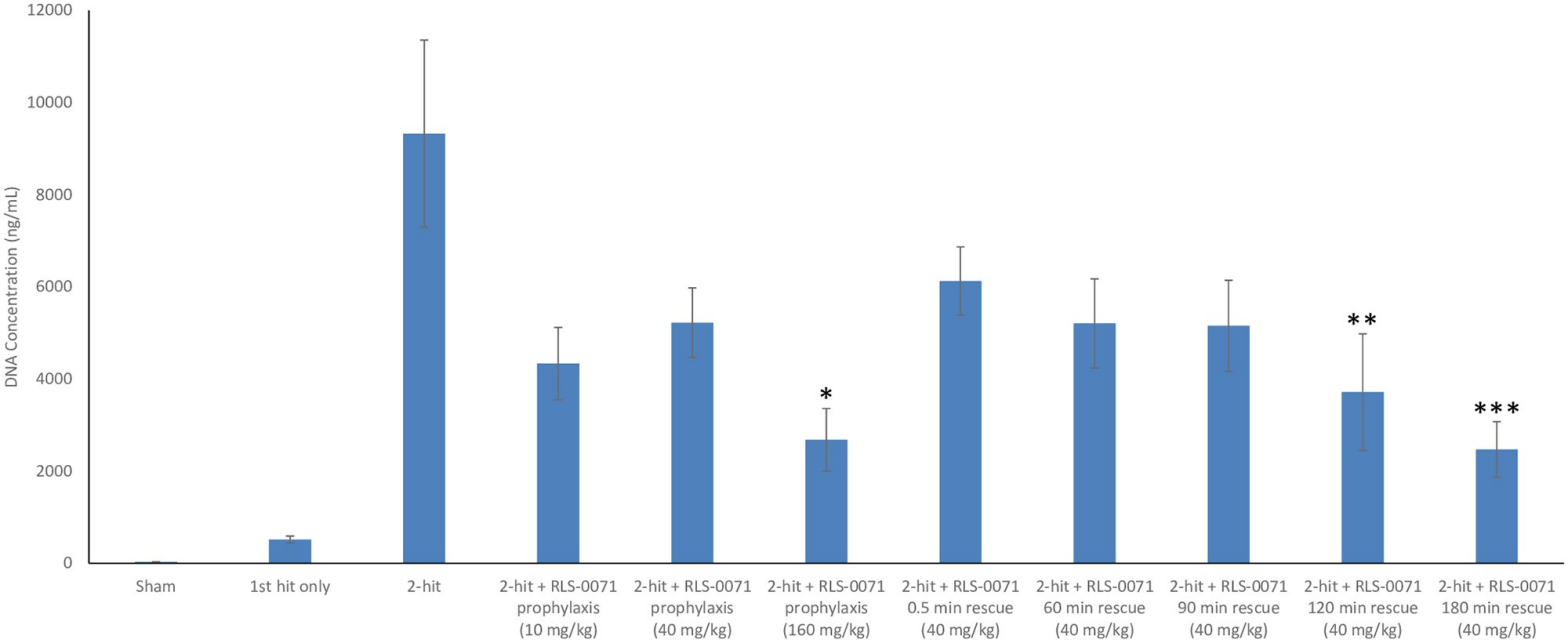
RLS-0071 reduces free DNA levels in the blood. Plasma was isolated from sham animals (n = 3) and the following groups at 4 hours after start of the experiments: first hit only (n = 3), 2-hit (n = 3), 2-hit + 10 mg/kg prophylactic dose RLS-0071 (n = 9), 2-hit + 40 mg/kg prophylactic dose RLS-0071 (n = 4), 2-hit + 160 mg/kg prophylactic dose RLS-0071 (n = 5), 2-hit + 40 mg/kg rescue dose RLS-0071 at 0.5 min (n = 4), 2-hit + 40 mg/kg rescue dose RLS-0071 at 60 min (n = 3), 2-hit + 40 mg/kg rescue dose RLS-0071 at 90 min (n = 5), 2-hit + 40 mg/kg rescue dose RLS-0071 at 120 min (n = 3) and 2-hit + 40 mg/kg rescue dose RLS-0071 at 180 min (n = 3). Plasma samples were incubated with PicoGreen. Fluorescence was read at an excitation wavelength of 485 nm and an emission wavelength of 520nm in a microplate reader. All free DNA measurements for each animal were done in triplicate. Data are means and standard error of the mean. Statistical analysis was performed using bootstrap approach or Welch’s ANOVA. *denotes p = 0.026, **denotes p = 0.039 and ***denotes p = 0.005 compared to 2-hit animals.

Neutrophil extracellular traps (NETs) released from activated neutrophils have been previously shown to play a pathogenic role in a variety of autoimmune, metabolic and inflammatory diseases [25]. NETs have been observed in murine models of virally induced ALI as well as TRALI and free DNA in the bloodstream is a biomarker for NETs in the blood of human patients with TRALI [15, 16] as well as COVID-19 patients [26]. We have previously shown that large amounts of free DNA are observed in our 2-hit model of ALI [14]. To ascertain the effect of RLS-0071 on free DNA levels in the blood, plasma from the different treatment groups were quantified in a PicoGreen assay 4 hours after transfusion. As expected, animals receiving the 2-hit insult showed high plasma levels of free DNA compared to sham animals and animals receiving the first hit of LPS only (Fig 5). Animals receiving the prophylactic doses of RLS-0071 showed reduced levels of free DNA at the 10 mg/kg, 40 mg/kg and 160 mg/kg doses with the 160 mg/kg dose demonstrating a significant reduction in free DNA compared to 2-hit only animals (p = 0.026). Animals subject to rescue doses of 40 mg/kg RLS-0071 after the second hit insult also showed reduced levels of free DNA when dosed up to three hours after the 2-hit injury with the rescue dosing at 120 and 180 minutes reaching statistical significance (p = 0.039 and p = 0.005, respectively). These results demonstrate that RLS-0071 can modulate NET formation in this disease model and this activity.

### RLS-0071 reduces inflammatory cytokine and chemokine levels in the blood

**Fig 6:**
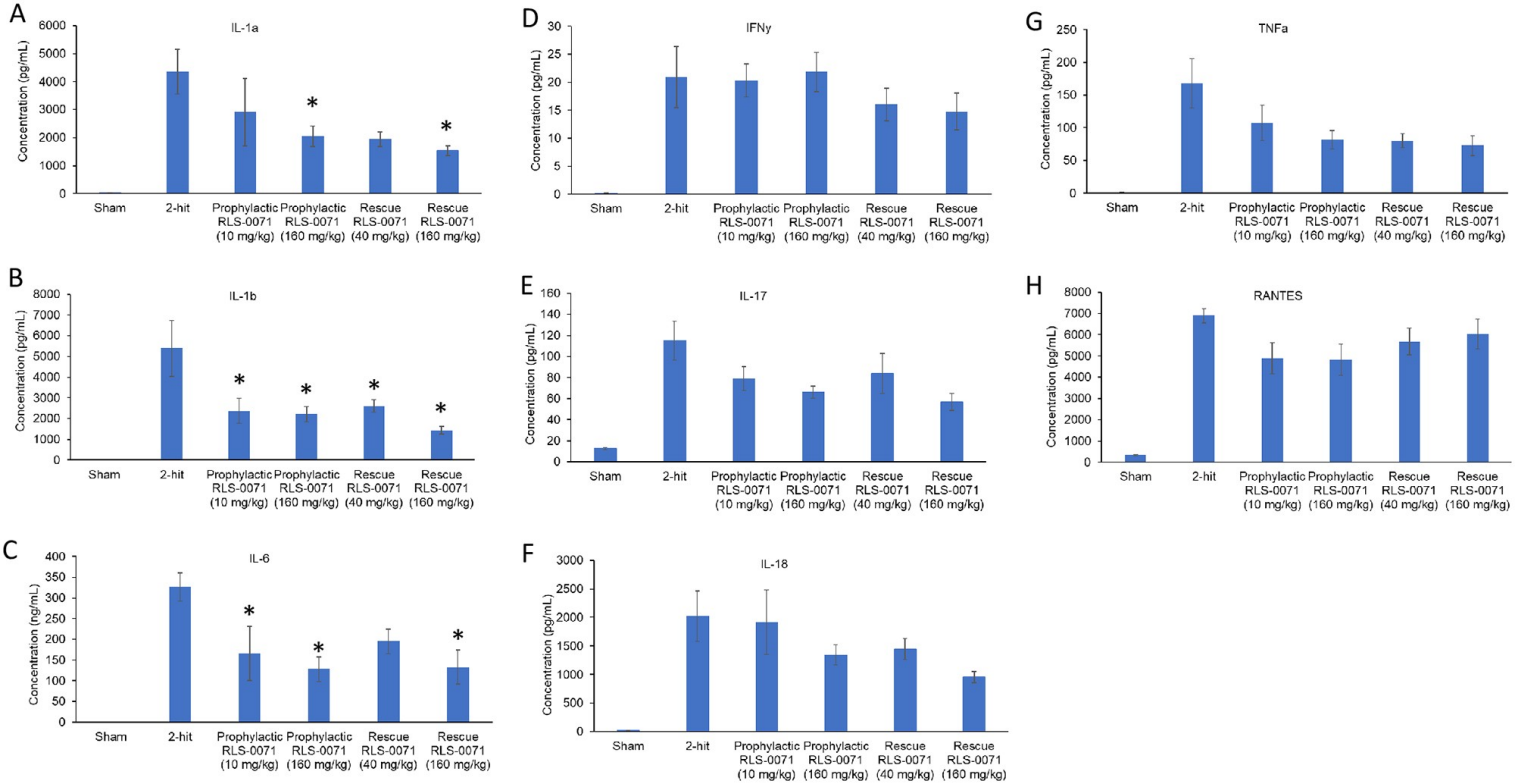
RLS-0071 reduces levels of inflammatory cytokines in the blood. Cytokine levels (A) IL-1a, (B) IL-1b, (C) IL-6, (D) IFN-g, (E) IL-17, (F) IL-18, (G) TNFa and (H) RANTES from terminal blood draws were determined by xMAP bead based immunoassay for the following experimental groups: sham, 2-hit, 2-hit + 10mg/kg prophylactic dose RLS-0071, 2-hit + 160mg/kg prophylactic dose RLS-0071 as well as 2-hit + 40mg/kg rescue dose RLS-0071 and 2-hit + 160mg/kg rescue dose RLS-0071. For sake of clarity only rescue dosing data is shown. Data are means and standard error of the mean. * denotes P <0.05 compared to animals receiving the 2 hit insult.

**Fig 7:**
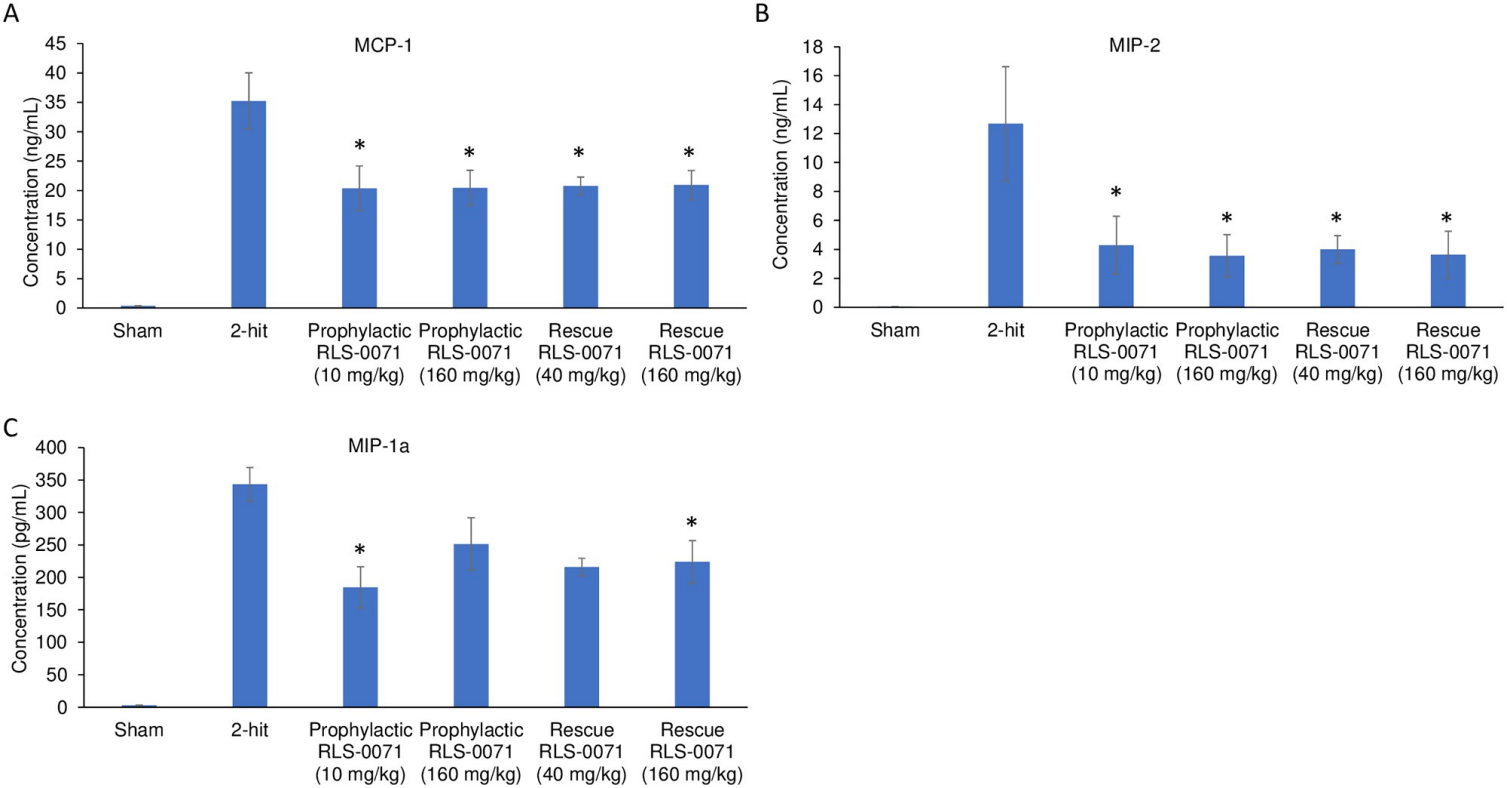
RLS-0071 reduces levels of inflammatory chemokines in the blood. Chemokine levels (A) MCP-1, (B) MIP-1a and (C) MIP-2 from terminal blood draws were determined by xMAP bead based immunoassay for the following experimental groups: sham, 2-hit, 2-hit + 10mg/kg prophylactic dose RLS-0071, 2-hit + 160mg/kg prophylactic dose RLS-0071 as well as 2-hit + 40mg/kg rescue dose RLS-0071 and 2-hit + 160mg/kg rescue dose RLS-0071. For sake of clarity only rescue dosing data is shown. Data are means and standard error of the mean. * denotes P <0.05 compared to animals receiving the 2 hit insult.

In severe cases of ALI, alveolar macrophages and epithelial cells may release significant amounts of pro-inflammatory cytokines that exacerbate the disease process leading to acute respiratory disease syndrome (ARDS). This so-called ‘cytokine storm’ has been well documented for virally-induced ALI, in particular the aggressive inflammatory response associated with severe outcomes in COVID-19 [27]. Given the significant ALI seen by lung histology in our rat 2-hit model, we measured the level of cytokines (Fig 6) and chemokines (Fig 7) from the blood of rats in absence or presence of RLS-0071 at the terminal 4 hour time point. As expected, plasma from sham animals had low levels of signal for all cytokines tested. Animals receiving the 1-hit of LPS only, had increased levels of cytokines whereas animals receiving the 2-hit insult had greater levels of cytokines which correlate with the increase in lung damage in the 2-hit animals as observed by histology (Fig 2). For each of the pro-inflammatory cytokines (IL-1a, IL-1b, IL-6, IFN-g, IL-17, IL-18, TNFa, and RANTES) and chemokines (MCP-1, MIP-1a and MIP-2) evaluated, animals receiving prophylactic dosing of RLS-0071 at 10 or 160 mg/kg and rescue dosing of RLS-0071 at 40 or 160 mg/kg had reduced levels of cytokines and chemokines compared to untreated 2-hit animals with some having significantly reduced levels (Figs 6 and 7). Taken together, these results demonstrate that a single prophylactic or rescue dose of RLS-0071 can mitigate severe ALI in this two-hit model through its dual inhibitory activity of complement inhibition and direct modulation of neutrophil-mediated NET formation.

## Discussion

The objective of this study was to determine if the anti-inflammatory molecule RLS-0071 was able to mitigate ALI in a novel 2-hit rat model that we have described previously [14]. The LPS first hit followed 30 minutes later with the incompatible erythrocyte second hit results in severe ALI within 4 hours after erythrocyte transfusion. As previously reported [14], the ALI observed by histology is mediated by robust neutrophil activation and sequestration into the lung tissue, classical and alternative complement pathway activation and as reported here, significant production of inflammatory cytokines. RLS-0071 is the lead derivative of the PIC1 family of compounds and has been demonstrated to inhibit classical complement activation in in vitro, in vivo and ex vivo studies and inhibit NET formation via inhibition of myeloperoxidase in in vitro and ex vivo studies [17–20]. Given the dual anti-inflammatory activities of complement inhibition and neutrophil modulation, we hypothesized that RLS-0071 would inhibit ALI in this animal model. Our results demonstrate that RLS-0071 delivered as a single dose either prophylactically or as a rescue dose was able to inhibit ALI even when delivered up to three hours after the second-hit of incompatible erythrocyte transfusion. This was demonstrated by reduced lung damage scores as assessed by histology, reduction of complement activation as measure C5a, decreased levels of free DNA which serves as a biomarker for NETosis and reduction of inflammatory cytokines and chemokines.

**Fig 8:**
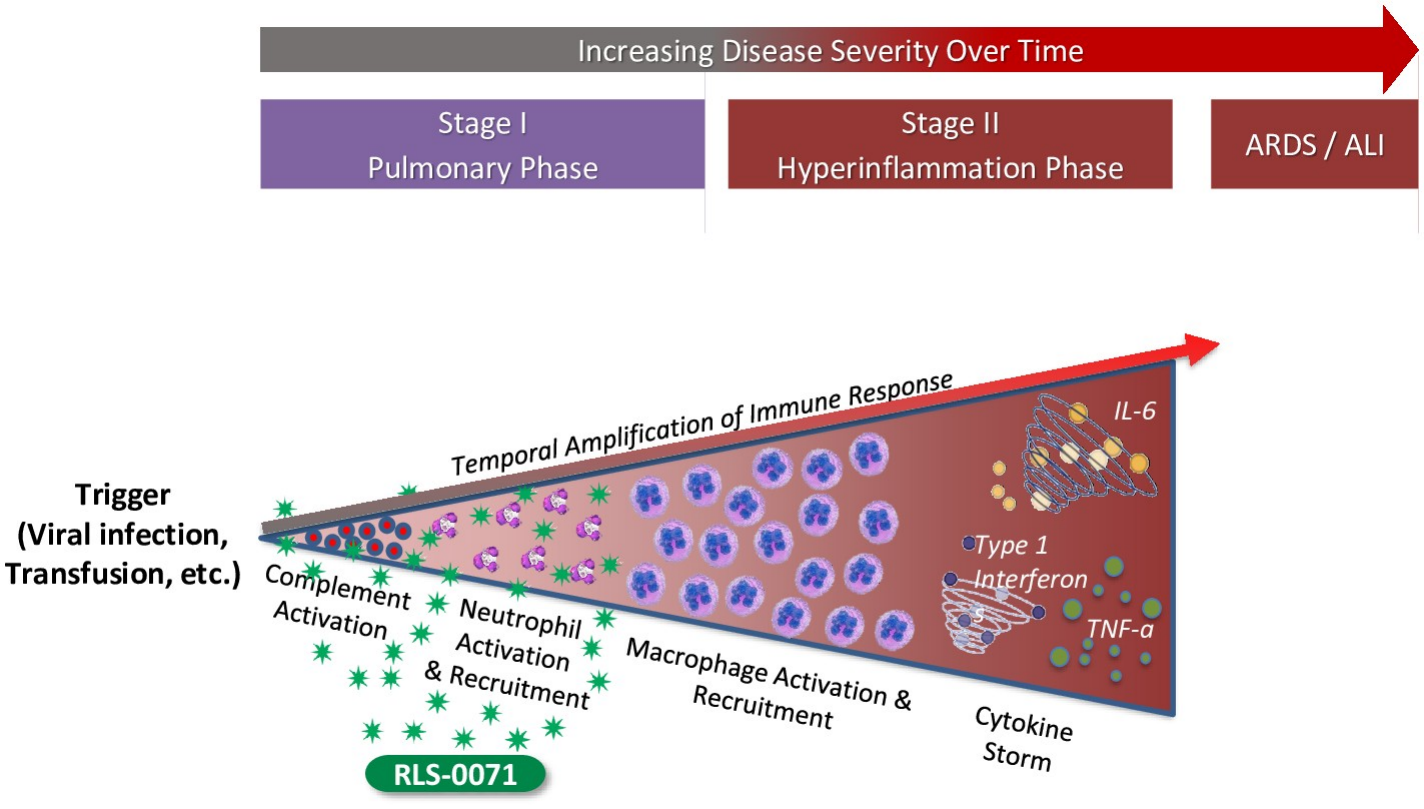
RLS-0071 inhibits ALI at the earliest stage of immune dysregulation. ALI ensues following triggering of the humoral (complement cascade) and cellular (neutrophil) innate immune response. RLS-0071, as illustrated by the green stars, downregulates both these processes by acting on the earliest stages of the inflammatory cascade preventing escalation of the cytokine storm and tissue damage.

ALI ensues following activation of the complement cascade and innate immune response by an external trigger such as a viral infection (e.g, COVID-19, RSV, or influenza) or transfusion and is influenced by the underlying health status of the patient. Complement activation occurs within seconds leading to neutrophil recruitment to the lung tissue and activation of these cells to produce NETs as well as recruit and activate macrophages which in turn produce inflammatory cytokines. This temporal amplification of the immune response leads to a hyperinflammation state that may progress to ALI/ARDS and death (Fig 8). The potent inhibition of ALI observed in this 2-hit model by RLS-0071 may be attributed to the dual anti-inflammatory activities of the molecule, namely complement inhibition and neutrophil modulation at the earliest stage of immune dysregulation. RLS-0071 can inhibit classical complement activity within 30 seconds of IV administration in the rat [17] and directly modulates neutrophil activation (NETosis and myeloperoxidase activity) [18–20]. By acting within seconds, RLS-0071 downregulates both the humoral and cellular aspects of the innate immune response at the earliest stage of the inflammatory cascade preventing the cytokine storm and ensuing tissue damage.

## Conclusions

This study demonstrates that a single dose of RLS-0071 given prophylactically or as a rescue therapy can modulate neutrophil-mediated ALI in this novel 2-hit rat model preventing ALI and inflammatory cytokine release. The ability of RLS-0071 to mitigate ALI in this two-hit model has potential for utility as a clinical therapeutic for virally induced ALI or TRALI. Additionally, it may have efficacy in other acute neutrophil-mediated pulmonary exacerbations characterized by a dysregulated immune response such as COPD or steroid-refractory neutrophilic asthma.

## Supporting information

S1 Data Excel spreadsheet containing minimal data sets for Figs 3–7. (XLSX)
